# Non-Linear Langevin and Fractional Fokker–Planck Equations for Anomalous Diffusion by Lévy Stable Processes

**DOI:** 10.3390/e20100760

**Published:** 2018-10-03

**Authors:** Johan Anderson, Sara Moradi, Tariq Rafiq

**Affiliations:** 1 Department of Space, Earth and Environment, Chalmers University of Technology, SE-412 96 Göteborg, Sweden; 2 Laboratory for Plasma Physics—LPP-ERM/KMS, Royal Military Academy, 1000 Brussels, Belgium; 3 Department of Mechanical Engineering and Mechanics, Lehigh University, Bethlehem, PA 18015, USA

**Keywords:** non-local theory, Lévy noise, Tsallis entropy, fractional Fokker–Plank equation, anomalous diffusion, 05.40Fb, 02.50Ey, 05.40-a

## Abstract

The numerical solutions to a non-linear Fractional Fokker–Planck (FFP) equation are studied estimating the generalized diffusion coefficients. The aim is to model anomalous diffusion using an FFP description with fractional velocity derivatives and Langevin dynamics where Lévy fluctuations are introduced to model the effect of non-local transport due to fractional diffusion in velocity space. Distribution functions are found using numerical means for varying degrees of fractionality of the stable Lévy distribution as solutions to the FFP equation. The statistical properties of the distribution functions are assessed by a generalized normalized expectation measure and entropy and modified transport coefficient. The transport coefficient significantly increases with decreasing fractality which is corroborated by analysis of experimental data.

## 1. Introduction

In magnetically confined (MC) plasma devices transport driven by turbulent fluctuations often severely limit the confinement time and thus impede the development of fusion as an alternative for electricity production. It is pertinent to understand and mitigate the effects of the turbulently driven transport where simplified models often are employed in order to elucidate the main features of the plasma turbulence. In magnetised plasmas, it is commonly accepted that turbulence is the primary cause of anomalous (i.e., elevated compared to collisional) transport [1,2]. It has been recognized that the nature of the anomalous transport processes is dominated by a significant ballistic or non-local component where a diffusive description is improper. The turbulence in MC tokamak plasmas is anisotropic in the parallel and perpendicular length scales to the magnetic field and taps free energy from the pressure gradient that can drive fluctuations in electrostatic potential and density [1,2]. The super-diffusive properties are often ubiquitously found in plasmas, such as the thermal and particle fluxes in the gradient region or in the Scrape-Off Layer (SOL) where the transport is dominated by the coherent structures (blobs) [3,4,5,6,7,8,9] and inherently possess a non-local character [10,11,12,13,14,15,16]. Moreover, there is a large quantity of experimental evidence that density and potential fluctuations measured by Langmuir probes at different fusion devices support the idea that these fluctuations are distributed according to Lévy statistics. This was illustrated for example in [4], where probability density functions (PDFs) of the turbulence induced fluxes at the edge of the W7-AS stellarator were shown to exhibit power law characteristics in contrast to exponential decay expected from Gaussian statistics. Furthermore, the experimental evidence of the wave-number spectrum characterized by power laws over a wide range of wave-numbers can be directly linked to the values of Lévy index α of the PDFs of the underlying turbulent processes. One widely used simplified model of a plasma is the Hasegawa–Wakatani model which was recently analysed by statistical methods in Reference [17]. It was concluded that even simplified models may have components of fractionality stemming from the non-linear interactions and the generation of large scale modes such as zonal and shear flows. The Hasegawa–Wakatani model allows for the electrons to dynamically and self-consistently determine the relationship between the density and the electrostatic potential through the turbulence. Moreover, fractal features in transport have been observed in many experiments in many different fields of research. In particular it has been found that there is strong evidence of non-local heat transport in JET plasmas [18]. In this paper, fractal features is synonymous to a system where power law statistics is found. Here it is important to keep in mind that, although a simplified fractional transport model is used, it indicates that there is a lack of physics in the current transport models based on the mean field theory, namely the super-diffusive character of heat transport. Finding a proper kinetic description of dynamical systems with chaotic behaviour is one of the main problems in classical physics [19,20,21,22,23,24,25,26,27,28,29,30]. Over the past two decades it has become obvious that behaviour much more complex than standard diffusion can occur in dynamical Hamiltonian chaotic systems. In principle, the orbits in dynamical systems are always theoretically predictable since they arise as solutions to simple system of equations such as Newton’s equations; however, these orbits are sensitive to initial conditions and thus very small changes in initial conditions may yield widely different outcomes. From the macroscopic point of view, the rapid mixing of orbits has been used as a motivation for assumptions of randomness of the motion and the random walk models [19]. In characterizing the diffusion processes in plasmas, the starting point is often Brownian motion where the mean value vanishes, whereas the second moment or variance grows linearly in time according to $\langle \delta x^{2}\rangle =2Dt$. However, taking into account the experimental data found in plasma experiments, it is evident that many phenomena exhibit anomalous diffusion where variance grows non-linearly in time such that $\langle \delta x^{2}\rangle =2Dt^{\alpha }$. The reason an anomalous diffusion approach is needed is due to the restrictive assumptions of locality in space and time, and lack of long-range correlations that is the basis of Brownian motion. There are two limits of interest for α where the first is super-diffusion with α>1 and the second is sub-diffusion with α<1. A super-diffusive description is most often appropriate for fusion plasmas. Lévy statistics describing fractal processes (Lévy index α where 0<α<2) lie at the heart of complex processes such as anomalous diffusion. Lévy statistics can be generated by random processes that are scale-invariant. This means that a trajectory will possess many scales, but no single scale will be characteristic and dominate the process. Geometrically, this implies the fractal property that a trajectory, viewed at different resolutions, will look self-similar. Such strange kinetics [19,24] may be generated by accelerated or sticky motions along the trajectory of the random walk [2]. Super-diffusivity may also occur as a result of variation in the step length of the motion, which breaks the assumption that a unique step length may, e.g., give rise to long-range correlations in the dynamics generated by the presence of anomalously large particle displacements connecting otherwise physically disjoint domains.

We note that, although sub-diffusive processes are beyond the scope of the present work, its properties have been studied in many different contexts where transport is often inhibited by sticky motion. Among sub-diffusive phenomena are holes in amorphous semiconductors, where a waiting time distribution with long tails has been introduced [31]. The sub-diffusive processes within a single protein molecule have been described by generalized Langevin equation with fractional Gaussian noise [32]. Turbulence and related anomalous diffusion phenomena have been observed in a wide variety of complex systems such as high energy plasmas, semiconductors, glassy materials, nanopores, biological cells, and epidemic proliferation.

The objective of the present paper is to explore the non-linear character of the fractional Fokker–Planck (FFP) equation resulting from a Langevin description driven by Lévy stochastic force with a non-linear interaction in the velocity. The present work is based on previous efforts reported in Reference [29] and may provide new insights on the recent developments in the modelling of the anomalous transport of charged particles in magnetised plasmas, such as the non-local heat transport found in JET plasmas.

The paper is organized in the following way: in Section 2, the model is presented, and the numerical results are shown and discussed in Section 3. The final section presents a discussion and conclusions.

## 2. The Fokker–Planck and Langevin Equations

Fractional kinetics is a powerful framework in describing anomalous transport processes exhibiting Lévy statistics. It is able to reproduce key aspects of anomalous transport including the non-Gaussian self-similar nature of the PDFs of particle displacement, and the anomalous scaling of the moments. It has been shown that the chaotic dynamics can be described by using the FFP equation with coordinate fractional derivatives as a possible tool for the description of anomalous diffusion [33]. Previous papers on plasma transport have used models including a fractional derivatives on phenomenological premises [6,34,35]. Additionally, the integro-differential nature of the fractional derivative operators allows the description of spatiotemporal nonlocal transport processes. In particular, in fractional diffusion, the local Fourier–Fick’s law is replaced by an integral operator in which the flux at a given point in space depends globally on the spatial distribution of the transported scalar and on the time history of the transport process. Using fractional generalizations of the Liouville equation, kinetic descriptions have been developed [36,37,38]. The currently applied model is based on the Langevin equation with a Lévy-stable noise term, where the applied noise exhibits a power law tail [39,40]. The generalized Central Limit Theorem for Lévy-stable processes is a particular weak-convergence theorem in probability theory. It expresses the fact that a sum of many independent and identically distributed (i.i.d.) random elements, or alternatively, random elements with specific types of dependence, will tend to be distributed according to one of a small set of attractor distributions. There are here two cases of special interest: the first is when the variance of the i.i.d. variables is finite and the attractor distribution is then a normal distribution, and the second is where the sum of a number of i.i.d. random elements with power law tail distributions decreasing as ${\left|x\right|}^{-\alpha -1}$ where 0<α<2 (therefore having infinite variance) will tend to a Lévy-stable distribution with a fractality index of α as the number of elements in the set increases.

The motion of a colloidal particle can be described by the Langevin equation in the case of Brownian motion and it will take the form

(1) $$\frac{d}{dt}v=-\nu v+A\left(t\right),$$

where *v* is the speed of the particle, −νv is the friction, and A(t) is the white stochastic force such that $\langle A\left(t\right)A\left(t^{\prime }\right)\rangle =2D\delta \left(t-t^{\prime }\right)$. Moreover, by assuming that A(t) is a Gaussian stochastic force, a Maxwellian velocity distribution may be obtained and would lead to the standard Fokker–Planck (FP) equation describing the evolution of the distribution function:

(2) $$\frac{\partial }{\partial t}P+v\frac{\partial P}{\partial r}+\frac{F}{m}\frac{\partial P}{\partial v}=\nu \frac{\partial }{\partial v}\left(vP\right)+D\frac{\partial^{2}P}{\partial^{2}v}.$$

Here *P* is the distribution function, *v* is the velocity, *F* is an external force, e.g., the electromagnetic force, *m* is the mass, ν is the friction, and *D* is the diffusion coefficient. The corresponding reduced FP equation, where the Lorentz force is neglected, to the Langevin equation is

(3) $$\frac{\partial }{\partial t}P=\nu \frac{\partial }{\partial v}\left(vP\right)+D\frac{\partial^{2}P}{\partial^{2}v},$$

which yields to the stationary state Maxwellian velocity distribution for P(v) [41,42]. However, if A(t) is a stochastic noise with the properties of a Lévy-stable process, the FP equation has to be modified in order to accommodate for power law tails of the form $P\left(v\right)\propto v^{-\alpha -1}$ for a Lévy stable with fractional index α. The FFP equation becomes

(4) $$\frac{\partial }{\partial t}P\left(v,t\right)=\nu \frac{\partial }{\partial v}\left(vP\left(v,y\right)\right)+D\frac{\partial^{\alpha }P\left(v,t\right)}{\partial^{\alpha }\left|v\right|}$$

where 0<α≤2 and |v|<∞. The time-dependent solution is readily found in the Fourier space where the fractional Riesz operator in 1 + 1D can be transformed to

(5) $$\frac{\partial }{\partial t}\hat{P}\left(k,t\right)=-\nu k\frac{\partial }{\partial k}\left(\hat{P}\left(k,t\right)\right)-D{\left|k\right|}^{\alpha }\hat{P}\left(k,t\right)$$

where the Fourier transformed distribution function can be determined to be

(6) $$\hat{P}\left(k,t\right)=\exp\left(-\frac{{D|k|}^{\alpha }}{\nu \alpha }\left(1-\exp\left(-\nu \alpha t\right)\right)\right).$$

The fractional Riesz derivative is defined through its Fourier transform ${}_{-\infty }{\hat{D}}_{x}^{\mu }f\left(x\right)=\frac{\partial^{\mu }f\left(x\right)}{\partial^{\mu }\left|x\right|}=-{\left|k\right|}^{\mu }f\left(k\right)$, see, e.g., [22] for more information. Here it should be noted that the time derivative only introduces a relaxation time dependent on the friction and the fractionality α, where a smaller α yield a longer relaxation time.

**Figure 1:**
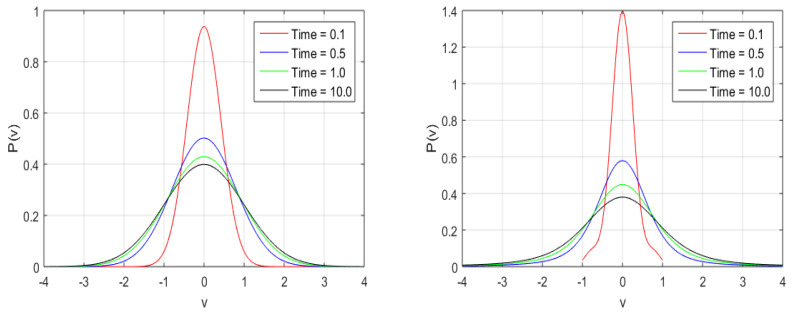
The probability density function (PDF) of velocity computed by the inverse Fourier transform of Equation (6) with α=2.0 (**left**) and α=1.5 (**right**) for t=0.1,0.5,1.0,10.0.

In Figure 1, the exponentially fast relaxation of the velocity PDFs with time is displayed. The PDFs of a Gaussian (α=2.0) and for a PDF with fractional index α=1.5 for times t=0.1,0.5,1.0, and 10.0 are computed numerically. We note that, at t=10.0, the PDFs are close to the stationary state PDF, whereas the time evolution of the PDF depends on the fractional index α such that the relaxation process is slower for a PDF with a lower fractional index. In general, the distributions found for the α=1.5 have more pronounced tails and sharper peaks, whereas, in the α=2.0 case, the system has a shorter relaxation time.

## 3. Results

The aim of the present paper is to look into the effects of a non-linear interaction in the Langevin equation, but it is here assumed that we can neglect the time dependence, i.e., the stationary state PDF ($\frac{dP}{dt}=0$), and the FFP Equation can be written as

(7) $$0=\nu \frac{\partial }{\partial v}\left((v+\beta v^{3})P\right)+D\frac{\partial^{\alpha }}{\partial^{\alpha }\left|v\right|}P.$$

Here ν, β, and *D* are constants. The equation is obtained by inclusion of the quartic potential, leading to the addition of a term of third order of the form $\beta v^{3}$. The main effect of retaining the temporal dynamics is to introduce a relaxation time. In the current model, square and quartic terms will be retained. The properties of the current non-linear terms are analogous to a potential well with square and quartic terms. Note that even terms in the potential provide proper stable equilibria, whereas odd terms yield an unstable equilibrium; thus, the third and fifth order terms are neglected. The Equation (7) is directly integrated by using a predictor—corrector method according to Adams-Bashforth-Moulton [43].

To find an analytical solution of the original Langevin equation, the Fourier transform can be used;

(8) $$\nu \left[\frac{\partial }{\partial k}-\beta \frac{\partial^{3}}{\partial k^{3}}\right]\tilde{P}+D{\left|k\right|}^{\alpha -1}\tilde{P}=0.$$

The found equation is a third order ordinary differential equation with variable coefficients. The general solutions to Equation (3) can only be determined by numerical means however a similar system was investigated in Reference [26] suggesting a PDF proportional to $exp(-a*v^{4})$, where *a* is a constant. Furthermore, it is also possible to find an analytical solution for the tail of the PDF to leading order by using the Wentzel–Kramers–Brillouin (WKB) approximation for small values of β. The WKB anzats is to assume a series solution to the Fourier transformed equation (3), of the form $\tilde{P}\left(k\right)=exp\left(\frac{1}{\epsilon }\sum_{n=0}^{\infty }\epsilon^{n}S_{n}\left(k\right)\right)$, here ϵ will be taken small and to be determined in terms of β. It is then found that, the leading order tail contribution corroborates the findings in [26] for α=2.0. We note that the real space distribution function is convergent for β>0 and can only in general be obtained by numerical integration, and is here solved by using method described above. We note that there are three different interesting regimes: the first is where the diffusion is much larger than the quartic potential strength $D/\nu >>\beta v^{2}$, the second is where the diffusion is comparable to the quartic potrential strength $D/\nu ∼\beta v^{2}$, and the third is where the diffusion is negligible to the quartic potential strength $D/\nu <<\beta v^{2}$. In the third regime, the PDFs become may be expected to have similarities to the results found in [26] for α=2.0 where $P\left(v\right)∼\exp\left(-av^{4}\right)$ for some constant *a*. The values used in this study are chosen to illustrate these three regimes of interest. Note that the non-linear interaction, i.e., the $\beta v^{3}$ term introduces three different regimes with richer dynamics which is in contrast to what was found in Reference [29]. In any linear fractal model based on the Lévy statistics the power law tails of the velocity PDF will be $P\left(v\right)\propto {\left|v\right|}^{-\alpha -1}$. Even more interestingly, in non-linear models the precise scaling of the PDF tails are still open.

**Figure 2:**
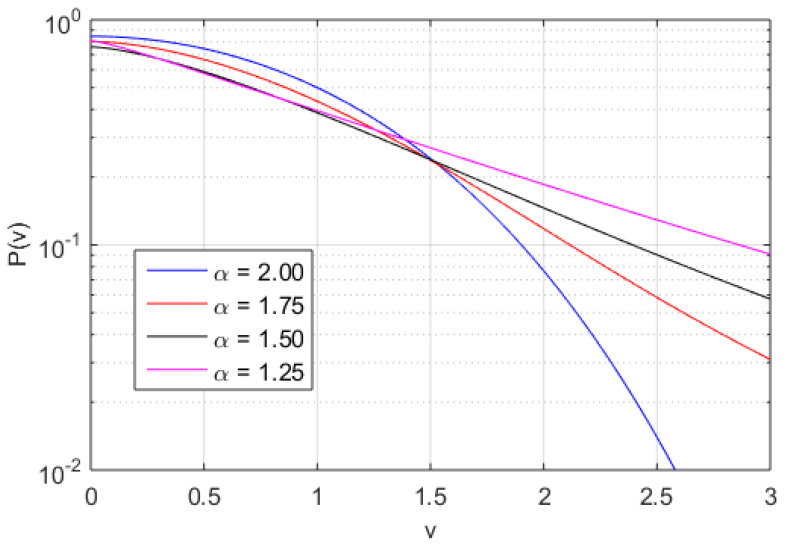
The PDF of velocity computed by integration of Equation (7) with with α=1.25 (magenta line), α=1.5 (black line), α=1.75 (red line), and α=2.0 (blue line) for D/ν=1.0 and β=0.1.

**Figure 3:**
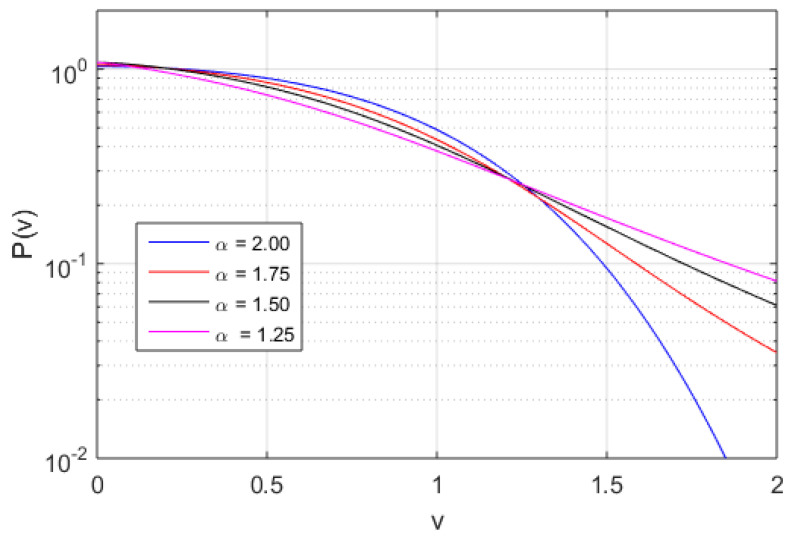
The PDF of velocity computed by integration of Equation (7) with α=1.25 (magenta line), α=1.5 (black line), α=1.75 (red line), and α=2.0 (blue line) for D/ν=1.0 and β=1.0.

**Figure 4:**
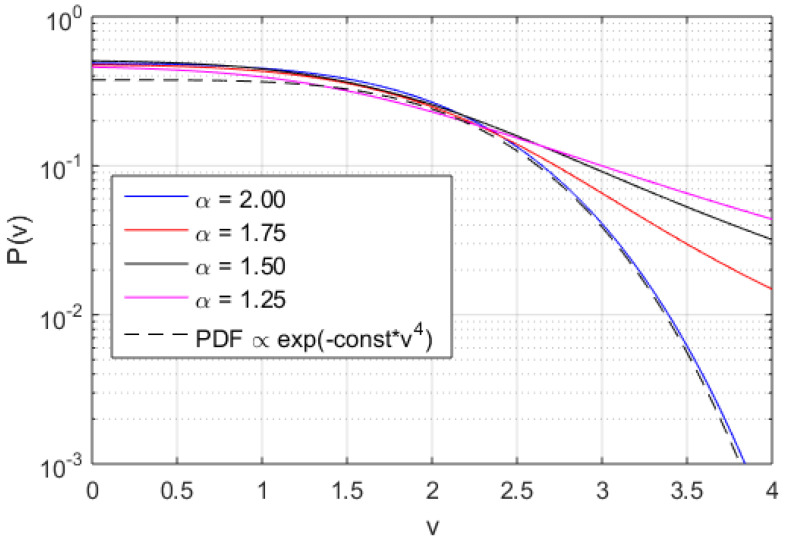
The PDF of velocity computed by integration of Equation (7) with with α=1.25 (magenta line), α=1.5 (red line), α=1.75 (red line), and α=2.0 (blue line) for D/ν=0.1 and β=1.0.

**Figure 5:**
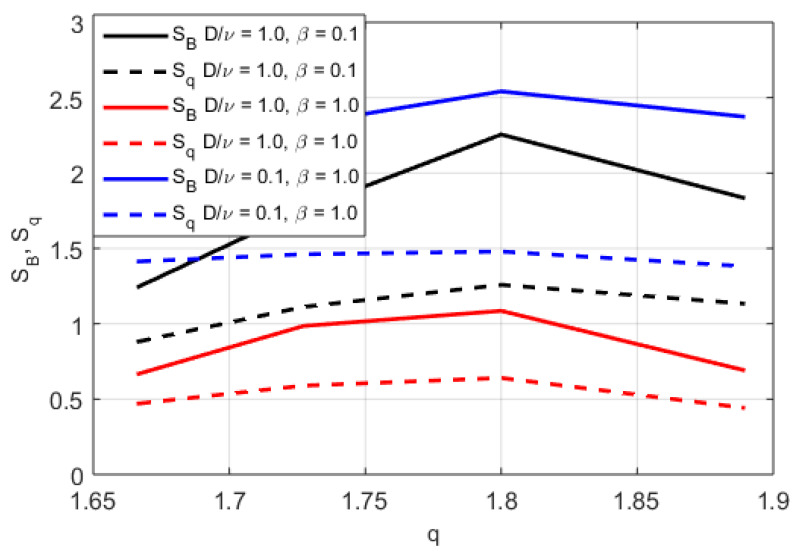
The Boltzmann–Gibbs entropy and the Tsallis’ entropy as functions of the fractality index *q* for D/ν=1.0 and β=0.1 (solid black line and dashed black line, respectively), D/ν=1.0 and β=1.0 (solid red line and dashed red, respectively), D/ν=0.1 and β=1.0 (solid blue line and dashed blue line, respectively).

**Figure 6:**
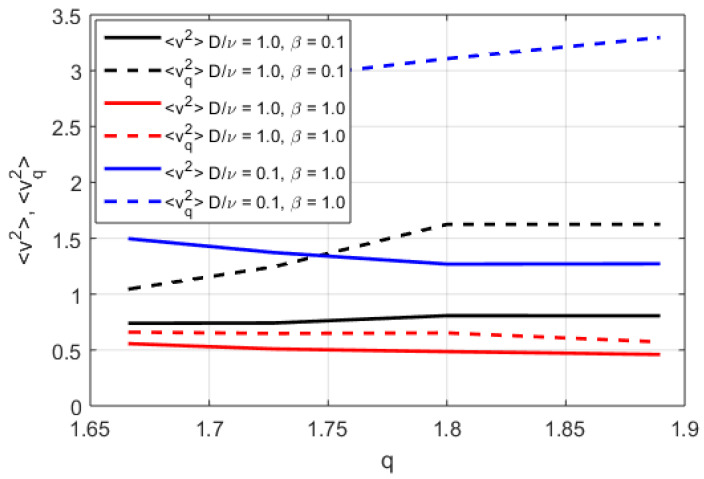
The energy and the generalized *q*-energy as functions of the fractality index *q* for D/ν=1.0 and β=0.1 (solid black line and dashed black line, respectively), D/ν=1.0 and β=1.0 (solid red line and dashed red, respectively), D/ν=0.1 and β=1.0 (solid blue line and dashed blue line, respectively).

In Figure 2, Figure 3 and Figure 4, the numerically found PDFs, by solving Equation (7) by the Adams-Bashforth-Moulton method [43], are shown for the three different regimes: D/ν=1.0 and β=0.1, D/ν=1.0 and β=1.0, and D/ν=0.1 and β=1.0, respectively. The resolution in *v* is $2^{-12}$ except in the case of α=1.25 for D/ν=1.0 and β=1.0 where the resolution is increased to $2^{-20}$, however increased resolution would not change the P(v) in any significant way for smaller |v|. As expected, in Figure 2 almost independently of α where $D/\nu >\beta v^{2}$, the PDFs exhibit power law tails, although in the case of α=2 some exponential behaviour is observed at the tail of the distribution (for large modulus of velocity |v|). In Figure 3 and Figure 4, we find more pronounced tails in particular in the low-α case. In the low-α case, the fractal term dominates the dynamics. We note that the PDFs displayed in Figure 3 exhibit a hybrid between fractal and Gaussian behaviour ($D/\nu ∼\beta v^{2}$), where in some cases the PDF is retains some Gaussian behaviour, which is particularly visible for small |v|. In the regime $D/\nu <<\beta v^{2}$ the non-Gaussian effects of the PDFs are clearly visible in Figure 4. The PDFs are used to evaluate the dynamics of the system in terms of Tsallis’ statistical mechanics where *q*-entropy and *q*-energy determines the properties of the system for the three different regimes: D/ν=1.0 and β=0.1, D/ν=1.0 and β=1.0, and D/ν=0.1 and β=1.0 in Figure 5 and Figure 6, respectively. The *q*, *q*-entropy, and *q*-energy values are determined by the following relations (see References [29,44,45,46,47,48,49,50,51,52,53,54]):

(9) $$\begin{array}{ccc} q & = & \frac{3+\alpha }{1+\alpha } \end{array}$$

(10) $$\begin{array}{ccc} S_{q} & = & \frac{1-\int dv{\left(F\left(v\right)\right)}^{q}}{q-1} \end{array}$$

(11) $$\begin{array}{ccc} {\langle v^{2}\rangle }_{q} & = & \frac{\int dv{\left(F\left(v\right)\right)}^{q}v^{2}}{\int dv{\left(F\left(v\right)\right)}^{q}}. \end{array}$$

The entropy and *q*-entropy are displayed in Figure 5. A maximum in the entropy, computed according to $S_{B}=\int dvP\left(v\right)lnP\left(v\right)$, is found, whereas almost constant *q*-entropy, computed according to Equation (10), is found with increasing *q*, where *q* is determined according to Equation (9). An increasing trend with importance of non-local effects are also visible where the entropy and *q*-entropy is increased in the cases with β=1.0, which is in the regime where the fractal nature is more prominent. In Figure 6, a decreased energy and increased *q*-energy with increasing *q* for β=1.0 and small D/ν case is found.

The interpretation of this strange kinetics has to be based on the results from experimental data since there is no first principle method to compute the value of α and thus *q* is indeterminable. However, recent findings suggest that JET plasmas have a significant degree of super-diffusive transport with an α<2, and it was found that this super-diffusive transport is slightly different for the ion and electron channels [18]. The analysis is based on a power balance where a large set of JET shots are used whereby a distribution in α can be obtained with a mean value of approximately 1, suggesting that a convective model would be more appropriate with q≈2. The diffusion coefficient can be estimated by the velocity autocorrelation functions according to the Kubo formula, but such an estimate looks at the ratio of the generalized diffusion coefficient ($D_{\alpha }$) and the Brownian diffusion coefficient $D_{0}=D\left(\alpha =2.0\right)$, using the tempered *q*-velocity correlators, computed by Equation (11).

**Figure 7:**
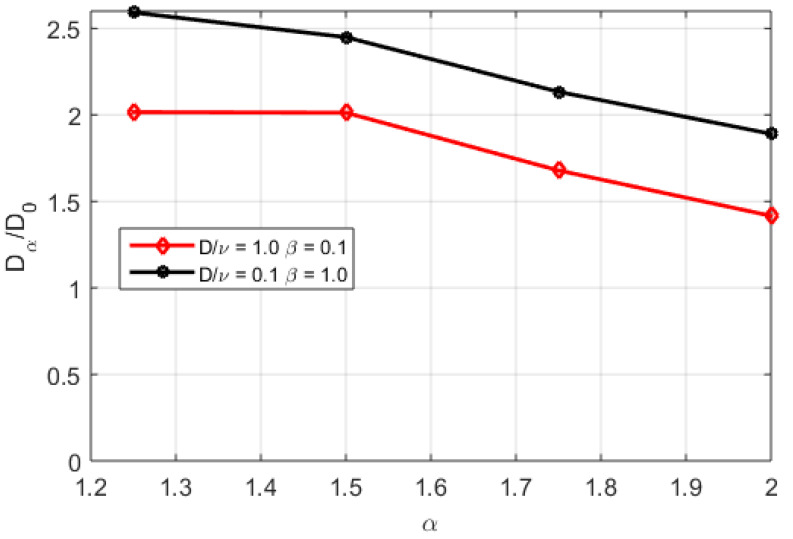
The ratio of the generalized diffusion coefficient ($D_{\alpha }$) and the Brownian diffusion coefficient as functions of the fractality index α for D/ν=1.0 and β=0.1 (red line) and D/ν=0.1 and β=1.0 (black line).

We find that the ratio of the diffusion coefficients increases with smaller α and significantly increases in the regime where fractality is pronounced, as shown in Figure 7. Interestingly enough, in the analysis presented in [18] it is evident that in the cases with increased transport a lower value of α is obtained, indicating a strong non-diffusive component or equivalently, a significantly increased transport where processes following Lévy statistics dominate the transport. The qualitative increase in the generalized transport coefficient $D_{q}$ is thus qualitatively corroborated by what is seen experimentally using the power balance analysis.

## 4. Discussion and Conclusions

Understanding anomalous transport in MC plasmas is an outstanding issue in controlled fusion research. It is commonly accepted that, in these plasmas, turbulence is the primary cause of anomalous (i.e., elevated compared to collisional) transport. It has also been recognized that the nature of the anomalous transport processes is dominated by a significant ballistic or non-local component where a diffusive description is improper. A satisfactorily understanding of the non-local features as well as the non-Gaussian PDFs found in experimental measurements of particle and heat fluxes is still lacking [25,26], but there has been some recent progress in this direction. Fractional kinetics has been put forward for building a more physically relevant kinetic description for such dynamics. In these situations, kinetic descriptions, which arise as a consequence of averaging over the well-known Gaussian and Poissonian statistics (for diffusion in space and temporal measures, respectively), seem to fall short in describing the apparent randomness of dynamical chaotic systems [19]. This is due to the restrictive assumptions of locality in space and time, and the lack of long-range correlations that is the basis of these descriptions.

In magnetised plasma experiments, a predator–prey system exists with avalanches (strong driver of transport) and zonal flows (sheared flows that decorrelate turbulent eddies reducing transport). It has been suggested that an Fractional Fokker–Planck Equation on a comb-like potential background can be applied where meso-scale transport events (avalanching) occurs in between regions of strong zonal flow activity (see Milovanov and Rasmussen [55]). This method is straightforward for applications in this setting; by assuming the used potential in between the zonal flow regions, it is suggested that the potential should be of degree 4 (or higher), as has been used here.

Although there has previously been some criticism on the appropriateness of using the Tsallis method in describing processes with Lévy statistics, this is mainly concerning descriptions based on fractality in coordinate space not in velocity space. However, the aim of the present work was to shed light on the non-extensive properties of the velocity space statistics and characterization of the fractal processes by estimating the generalized diffusion coefficients of the FFP equation in terms of Tsallis statistics. Jespersen et al. [56] showed an example of the Langevin equation with a harmonic potential, and the Tsallis *q*-statistics had limited usefulness. The reason for this is that, using the variational calculus of Equation (10) with the appropriate constraints, the relation between α and *q* is $\alpha =\frac{4-2q}{q-1}$, which is different from Equation (9) and thus cannot reproduce the correct scaling. They then concluded that the Tsallis entropy was not the appropriate framework for Lévy flights in a harmonic potential described by the generalized FP equation. However, this limitation seems not to impede the usefulness of the application of Tsallis entropy on this Langevin equation where the correct scaling is obtained.

In summary, we have employed an FFP equation to find the PDFs and studied the *q*-entropy and *q*-energies in this system with a non-linear interaction in the FFP equation. We found a significantly elevated diffusion coefficient, which is qualitatively similar to what was expected in light of the analysis of the experimental data.
